# Right ventricular dysfunction in Chagas' Cardiomyopathy. Primary involvement or a resonant manifestation of Left ventricular dysfunction?

**DOI:** 10.1186/1532-429X-16-S1-P301

**Published:** 2014-01-16

**Authors:** Henrique T Moreira, Gustavo J Volpe, Henrique S Trad, Marcel Koenigkam Santos, Minna D Romano, Antonio T Pazin-Filho, Benedito C Maciel, José A Marin-Neto, Andre Schmidt

**Affiliations:** 1 Medical School of Ribeirao Preto, University of São Paulo, Ribeirao Preto, São Paulo, Brazil

## Background

Chagas' Cardiomyopathy (CC) is an important cause of heart failure and sudden cardiac death (SCD) in Latin America. Migratory movements have spread carriers throughout the world, especially in USA and Europe. Left Ventricle (LV) involvement is a landmark and apical aneurysm a pathognomical sign. Nevertheless, early involvement of the right ventricle (RV) and RV systolic dysfunction in presence of normal left ventricular systolic function in CC has been a subject of controversy in the literature. Therefore, we analyzed a cohort of patients with CC in order to explore the presence of RV dysfunction.

## Methods

125 patients with CC were submitted to cardiac magnetic resonance during an investigational protocol for primary prevention of SCD. SSFP short-axis cines were used to establish RV and LV volumes, mass and function. Late Gadolinium Enhanced (LGE) short axis sequences were visually analyzed to establish the presence of a LV scar. RV and LV systolic dysfunction were established using most recent recommendations of the European Society of Cardiology. Parametric tests and Chi-square tests were used as appropriate and P < 0.05 was established as significant.

## Results

Mean age was 55 ± 13 yo and 57% (71) were men. 44% had no ventricular dysfunction, 33% presented isolated LV dysfunction, 18% presented both RV and LV dysfunction and 5% had isolated RV dysfunction. Scar was detected in 77% of CC patients. Presence of a scar was associated with larger LV end systolic (119 ± 85 vs 66 ± 53 mL) and diastolic (201 ± 91 vs 144 ± 59 mL) volumes and mass (130 ± 36 vs 102 ± 30 g) and lower LV ejection fraction (45 ± 15 vs 59 ± 15%)- P < 0.05 for all. On the other hand, a relation between the presence of a LV scar and RV dimensions or function couldn't be established.

## Conclusions

CC can present any combination of ventricular dysfunctions. Although RV systolic dysfunction occurs most frequently in association with LV systolic dysfunction, notably RV dysfunction can be isolated and thus the presence of this finding should raise Chagas' Disease as a possible diagnosis. Presence of a LV scar indicated worst LV function and larger LV volumes but was not related to signs of RV dysfunction.

## Funding

None.

